# The contribution of age-related changes in the gut-brain axis to neurological disorders

**DOI:** 10.1080/19490976.2024.2302801

**Published:** 2024-01-18

**Authors:** Romeesa Khan, Claudia M. Di Gesù, Juneyoung Lee, Louise D. McCullough

**Affiliations:** a Department of Neurology, McGovern Medical School, The University of Texas Health Science Center at Houston, Houston, TX, USA; b University of Texas MD Anderson Cancer Center UTHealth Graduate School of Biomedical Sciences, Houston, TX, USA

**Keywords:** Microbiome, gut-brain axis, aging, maternal microbiome, neurodegeneration, neurological disorders, microbial metabolites, blood-brain barrier

## Abstract

Trillions of microbes live symbiotically in the host, specifically in mucosal tissues such as the gut. Recent advances in metagenomics and metabolomics have revealed that the gut microbiota plays a critical role in the regulation of host immunity and metabolism, communicating through bidirectional interactions in the microbiota-gut-brain axis (MGBA). The gut microbiota regulates both gut and systemic immunity and contributes to the neurodevelopment and behaviors of the host. With aging, the composition of the microbiota changes, and emerging studies have linked these shifts in microbial populations to age-related neurological diseases (NDs). Preclinical studies have demonstrated that gut microbiota-targeted therapies can improve behavioral outcomes in the host by modulating microbial, metabolomic, and immunological profiles. In this review, we discuss the pathways of brain-to-gut or gut-to-brain signaling and summarize the role of gut microbiota and microbial metabolites across the lifespan and in disease. We highlight recent studies investigating 1) microbial changes with aging; 2) how aging of the maternal microbiome can affect offspring health; and 3) the contribution of the microbiome to both chronic age-related diseases (e.g., Parkinson’s disease, Alzheimer’s disease and cerebral amyloidosis), and acute brain injury, including ischemic stroke and traumatic brain injury.

## 1. Introduction

Over the past decade, research has identified a novel role of the gut microbiome in the bidirectional communication between the gut and the brain, termed the microbiota-gut-brain-axis (MGBA).^1^ Disruption in the balance of gut microbial communities (often referred to as “dysbiosis”) has implicated various pathways along this axis that contribute to the progression of neurodegenerative diseases, such as Alzheimer’s disease (AD) and Parkinson’s disease (PD).^1^ The MGBA also contributes to outcomes after acute neurological injury, such as stroke and traumatic brain injury (TBI).^1^ Many of these NDs are diseases that increase in prevalence with aging. Emerging studies have shown that the process of aging directs and changes the composition of the microbiome,^2^ which leads to chronic systemic inflammation, or “inflammaging”.^3^ Inflammaging is characterized by an increased level of circulating pro-inflammatory cytokines and breakdown of the barrier integrity of host tissues including in the brain (e.g., blood-brain barrier) and gut (e.g., intestinal epithelium), leading to antigen translocation into the host and heightened systemic inflammation.^2,4^ Inflammaging may be caused by a variety of processes that accompany aging, such as oxidative stress or cellular senescence, many of which are mediated by recently identified pathways in the MGBA.^5^ In experimental studies, specific microbially-derived metabolites are altered both with aging and in age-related diseases, which has now been confirmed in clinical populations as well.^6^ Our understanding of the mechanisms through which these metabolites change host homeostasis is emerging, but detailed mechanistic studies are required if we hope to harness the potential of the microbiome to enhance health.

## 2. The microbiome as a mediator in gut-to-brain signaling

The human microbiome is comprised of trillions of microorganisms, including bacteria, archaea, viruses, and eukaryotes, which exert a profound effect on all physiological and pathological processes occurring in the host.^1,7^ Microbial communities vary across distinct body sites and organs, but those residing in the gastrointestinal (GI) tract have, to date, attracted the greatest attention in biomedical research, including gerontology and neurological research.

Multiple large-scale studies, such as the NIH-funded Human Microbiome Project (HMP), have provided extensive information regarding the genetic sequences of most microorganisms residing in the human gut. These datasets revealed that the phyla *Bacteroidetes* and *Firmicutes* account for 90% of the gut microbiota, but many others such as *Proteobacteria*, *Actinobacteria*, *Fusobacteria*, *Spirochaetes*, *Verrucomicrobia* and *Lentisphaerae* are also present. The identification of a core microbiome, despite being critical for our understanding of the microbial contribution to health and disease, is challenging due the huge variability in gut microbiome configurations across individuals and within the same subject across the lifespan.^8^ More recently, multi-omics approaches such as metagenomics and metabolomic sequencing has dramatically expanded our knowledge of the functional role of gut microorganisms in host health and pathological processes.^9^ The interplay between the gut microbiota and the host is regulated by a complex network of metabolic, immune, and neuroendocrine interactions. When physiological changes within the gut microbial community evolve into a detrimental state, or so-called “dysbiosis”,^10^ significant alterations in the pool of the metabolites produced and released by these microorganisms occur, with important repercussions for host physiology.

A growing body of evidence has implicated the gut microbiome in the progressive accumulation of molecular and cellular alterations observed with senescence. These changes ultimately increase the susceptibility to chronic diseases in older populations. Aging-associated processes include the progressive accumulation of senescent cells (SCs), which are identified by definitive cell cycle arrest, abnormal mitochondrial reactive oxygen species (ROS) production, metabolic shifts, and the production of senescence-associated secretory phenotype factors (as reviewed in ref^11^). Release of these factors triggers proinflammatory responses from different immune cells that participate in the physiological changes seen with aging. These detrimental changes are potentially reversible, as shown by recent studies in mice that target these SCs, leading to reductions in systemic inflammation, TNFα/NF-κB signaling, and senescence-associated signatures in aged mice.^12^ Intriguingly, emerging evidence suggests that the gut microbiome may play an important role in modulating the effects of SC. A very recent study using germ-free (GF) mice (raised in total absence of microbial colonization) showed that the aging microbiome was responsible for accumulation of senescent markers in ileal B cells, which in turn further altered gut microbiome composition.^13^

## 3. The gut-brain axis: a bidirectional communication

**Figure 1.**
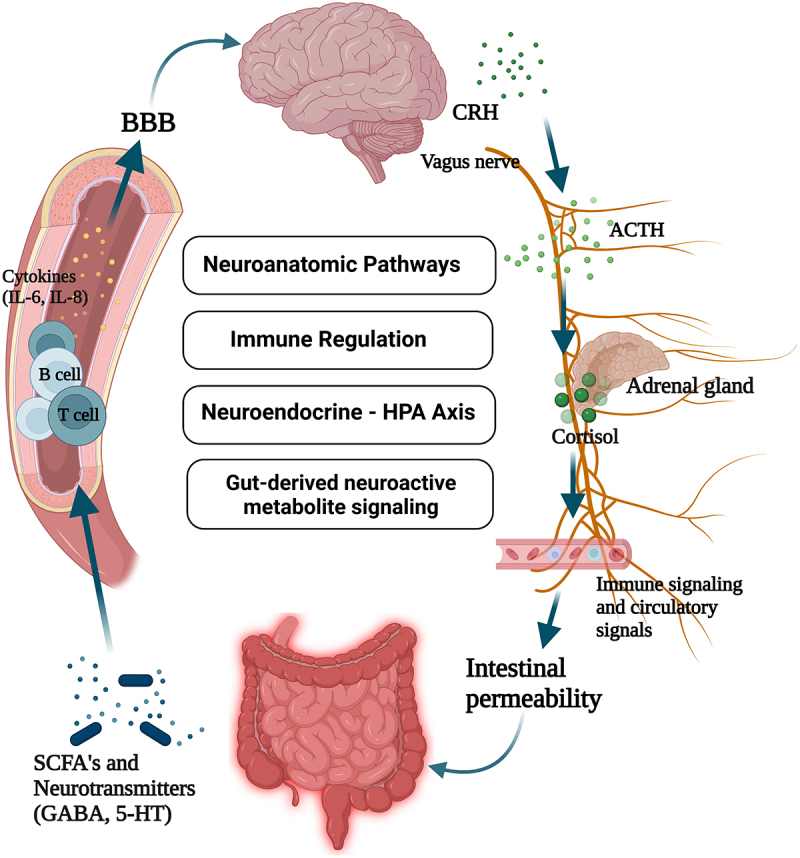
The bidirectional communication between the gut and central nervous system is regulated by multiple pathways(BBB = blood brain barrier, CRH = corticotropin-releasing hormone, ACTH = adrenocorticotropic hormone, HPA = hypothalamic-pituitary-adrenal, GABA = gamma-aminobutyric acid, SCFA = short chain fatty acid, 5-HT = 5-hydroxytryptamine). Created in Biorender.com.

Communication between the brain and microbiota is bidirectional and can occur through multiple pathways. These include neural connections between the brain and the gut through the vagus nerve, hormonal and immune pathways, and metabolite signaling.^14^ There is a high degree of intercommunication between the gut and the peripheral nervous system (PNS) which participate in the immunological and hormonal responses to gut bacterial biochemical processes. Gut microbial signals can be “sensed” via vagal and spinal neurons, integrated in the brainstem and hypothalamus, and ultimately influence efferent signals to peripheral organs.^14^ Several recent studies manipulating the gut microbiota composition have illustrated the importance of the interaction between gut microbes and the PNS (Figure 1), via efferent/afferent pathways, in regulating host physiology, as discussed below.

### 3.1 Neuroanatomic pathways: vagal mechanisms/vagus nerve

**Figure 2.**
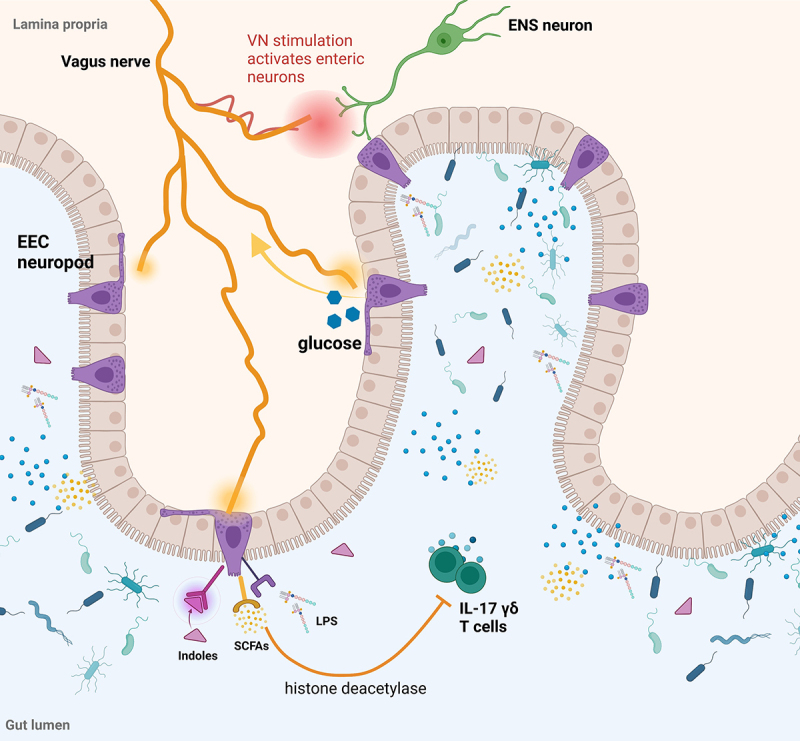
Vagus nerve interactions with the gut epithelium and the ENS. Created in Biorender.com.

The two-way neuroanatomic communication between the gut and brain occurs through afferent or efferent signaling along two main directional pathways: (1) the autonomic nervous system (ANS) including the vagus nerve (VN) and the enteric nervous system (ENS). The VN is the tenth cranial nerve and one of the main components of the parasympathetic nervous system, which forms the ANS together with the sympathetic nervous system. The ANS has a primary role in regulating multiple physiological processes, including heart rate, immune response, and digestion.^15^ Signals from the gut are conveyed to the central nervous system (CNS) through the ANS in a bottom-up manner, and responses from the CNS are then sent to the gut by the ANS in a top-down manner.^16^ In this context, the VN represents the most direct connection between the gut and the brain, participating in both bottom-up and top-down signaling via both afferent (sensory) and efferent (motor) nerves.^17^ Vagal terminals reach the gut in the mucosal layer, the smooth muscle layer, and synapse with enteroendocrine cells (EECs), without direct contact with the gut microbiota in the lumen. A recent study by Bohórquez and colleagues^18^ showed the presence of a specialized group of EECs, defined as neuropods, which provide a direct connection between the gut lumen and brain stem by synapsing with the VN through glutamatergic transmission. The neuropod-vagal terminal circuit is activated in response to sugar, thereby transducing fast sensory input from the gut lumen. Additionally, vagal fibers, which express receptors for multiple metabolites produced by the microbiome, can sense changes in microbial populations.^19^ Similarly, EECs express receptors for microbial metabolites such as short-chain fatty acids (SCFAs), indoles, bile acids, and lipopolysaccharide (LPS).^20^ Both human and animal studies have highlighted the crucial role of the VN in regulating brain activity. Either partial or total vagotomy in rodents led to changes in brain circuits and behavioral functions implicated in various neuropsychiatric disorders,^21^ such as anxiety, fear-related phenotypes,^22^ learning and memory,^23^ locomotion^24^ and sensorimotor gating.^25^ Similarly, direct stimulation of the VN modulates stress-induced depressive phenotypes via regulation of serotonergic circuitry in the hippocampus,^26^ in anxiety and post-traumatic stress disorders^27,28^ and the reward system involved in affective disorders through effects on dopaminergic circuitry in the substantia nigra.^29^ Recent studies have identified specific microorganisms residing in the gut that can modulate brain function via vagal fibers. For example, administration of *Lactobacillus rhamnosus* was effective in decreasing both depressive- and anxiety-like phenotypes in mice, an effect that was mediated by an increased firing rate of VN terminals.^11^ Similarly, supplementation with *Campylobacter jejuni* increased VN c-Fos expression in the vagal ganglia, leading to activation of neurons in the solitary nucleus of the brainstem.^30^ Additionally, another study showed that *C. jejuni* treatment in mice promoted anxiety-related behavior.^31^ The interactions between the vagus nerve, the gut epithelium and the ENS are summarized in Figure 2.

### 3.2 Neuroanatomic pathways: the enteric nervous system

The ENS, a part of the PNS, is at a critical intersection between the host and the gut microbiome. Anatomically, the ENS is organized as a web of motor, sensor, and interneurons that are embedded in the inner and outer layers of the muscularis externa and in the submucosa of the digestive system. By integrating peripheral sensory information with input from the ANS, the ENS controls the muscular and secretory functions of the GI tract, including peristalsis and the production and release of enzymes and hormones, such as gastrin and secretin.^28^ Sensory neurons in the ENS form synapses with both enteric motor neurons and vagal fibers, and express receptors for multiple microbial metabolites and components, including SCFAs^32^ and LPS through toll-like receptor 4 (TLR4).^33^ Studies in GF mice have shed light on the influence of the microbiome in controlling the electrophysiology of ENS neurons. Maturation of the ENS begins during postnatal development as microbial strains colonize the infant gut through the activation of pattern recognition receptors, such as TLRs, on ENS terminals that bind to microbial products, including LPS.^34^ Microbial reconstitution of GF mice induced upregulation of the expression of 5-hydroxytryptamine (5-HT) and its receptors in enteric neurons.^34,35^ Antibiotic-mediated depletion of gut bacteria in mice altered both the morpho-functional structure and the neurochemistry of the ENS, including the loss of neurons in the myenteric plexus, increased TLR2 expression in neuromuscular and mucosal layers in the ileum, and a reduction in glial cells.^36^ Different microorganisms may exert different effects on ENS neuronal activity through distinct mechanisms.^37^ Recent studies suggest that changes in the ENS might be a driving factor in determining dysbiosis of the gut microbiome through the regulation of intestinal transit, gut barrier permeability, and luminal pH.^38^

### 3.3 Systemic and mucosal immune regulation: immunological pathways

Studies in both animal models and humans have shown that the gut microbiome is essential for the regulation of the host immune system. Microbial colonization of the host’s mucosa during the early postnatal period profoundly shapes the development and maturation of the host immune system.^39^ Beyond infancy, the gut microbiome is intricately involved in maintaining immune homeostasis through complex interactions with the mucosal immune system. Immune cells also play important roles in the gut^40^ including: (1) tolerance toward a multitude of microorganisms in the normal, healthy gut ecology; (2) surveillance of potential pathogenic strains; and (3) inhibition of commensal overgrowth and prevention of translocation of bacteria from the intestinal lumen into the host, a process that requires the integrity of the intestinal mucosal barrier.^41^

Crosstalk between the gut microbiota and host immune system regulates the production and release of neurotransmitters and neuropeptides, cytokines, and other signaling mediators that influence brain function by multiple mechanisms, including actions on vagal and spinal afferent fibers.^42^ In this context, one of the mechanisms of regulation of the MGBA involves the maturation and function of microglia, the resident immune cells of the brain.^42,43^ This process starts during fetal and early postnatal development and is mediated by the composition of the maternal gut microbiome.^44^

During prenatal development, maternal gut microbial communities contribute to fetal microglia programming, which in turn, directly affect the formation of cortical cytoarchitecture and neural circuits.^45^ In GF mice, substantial alterations in gene expression occur in fetal microglial at mid and late gestation. Microglia programming continued postnatally (P20) leading to increased microglial density (Iba1^+^ cells) in the somatosensory neocortex of GF females compared to SPF females, which was not seen in males.^44^ The impaired microglia maturation seen in GF mice was rescued by early-life colonization with a complex microbiome.^46^ Migration of CD4 T cells into the brain around the time of birth is also critical for microglial maturation, and is potentially regulated by the gut microbiome.^47^ Therefore, further studies are needed to investigate how alterations in the gut microbiome composition and immune cell populations in early development could lead to increased predisposition for neurological disorders later in life.

Microglia not only modulate inflammatory processes in the brain, but are also involved in synaptic plasticity and remodeling, maturation of the CNS, and debris and aggregate clearance.^48^ Microglia activation and function can be mediated by several factors produced from host-bacteria interactions,^49^ including cytokines, tryptophan metabolites, bacterial-derived cell components (e.g., peptidoglycans and LPS)^50^ and bacterial-derived metabolites (SCFAs).^51^ These gut-derived signals can reach the brain through the bloodstream, through the VN^52^ and potentially through the newly discovered meningeal lymphatic system through actions on γδ T cells.^53^ Several studies have proposed epigenetic regulation and chromatin remodeling as a potential mechanism for microbiota-dependent modulation of microglia function.^43,54^ A recent investigation in the context of neurodegenerative processes^55^ identified SCFAs, specifically acetate, as a crucial regulator of microglial metabolic function through histone methylation modification on genes related to microglial proliferation, morphology, activation, and metabolism. Epigenetic regulation of immune function via bacterial metabolites has also been observed in the gut immune system. For example, SCFAs, particularly propionate, decreased IL-17-producing γδ T cells in humans (e.g., peripheral blood mononuclear cells) and in mice (e.g., intestinal lamina propria) in a histone deacetylase-dependent manner.^56^ Further research is warranted to unravel the precise mechanisms by which the gut microbiome modulates microglia and, as such, brain function. However, these studies have provided mechanistic insights that might lead to the identification of new microbiome-based therapeutic strategies that target microglial function in brain disorders.

### 3.4 Neuroendocrine-hypothalamic-pituitary-adrenal axis pathway

The neuroendocrine system regulates many processes in the human body and plays a critical role in organ development and function. Among the primary neuroendocrine pathways, the hypothalamic – pituitary – adrenal (HPA) axis is thought to be tightly connected to the gut microbiome in the context of the MGBA. The HPA includes the hypothalamus, the pituitary gland, and adrenal glands.^57^ In the context of the stress response, the hypothalamus receives stimuli to produce and release corticotrophin-releasing hormone (CRH), which induces the secretion of adrenocorticotropic hormone (ACTH) from the pituitary gland. ACTH stimulates the adrenal cortex to release glucocorticoids, mineralocorticoids, and catecholamines, which then modulate other downstream processes to produce appropriate responses to the stressor.^58^ Bidirectional communication between the neuroendocrine-HPA axis system and the gut microbiota involves multiple other components of the MGBA, including the immune system, gut hormones and microbially-derived products, as well as receptors expressed on both the intestinal and the blood-brain barriers as reviewed in ref.^58^ Several studies have suggested that microbially-derived compounds, such as precursors of neurotransmitters and gut hormones, and SCFAs, can regulate the neuroendocrine system.^58,59^ Similarly, the microbiome may participate in HPA axis modulation through the immune system.^60^ Hyperactivation of the HPA axis is associated with disorders affecting both the neuroendocrine system and the gut microbiome, such as irritable bowel syndrome (IBS) and depression. One intriguing hypothesis^61^ suggests that HPA axis dysregulation might lead to gut dysbiosis and alterations in the integrity of the intestinal barrier, which in turn could promote chronic low-grade inflammation that is seen in both IBS^62,63^ and depression.^64,65^

The microbiome is involved in the bidirectional communications between the neuroendocrine system, including the HPA axis, and the immune response^66,67^ as reviewed in ref.^68^ The integrity of the intestinal barrier can be altered by neuroendocrine mediators released in the context of stress response, thus facilitating the release of microbially-derived molecules, which subsequently activate immune pathways.^69^ For instance, levels of bacterial LPS increase the production of cytokine colony-stimulating factor 1 in muscularis macrophages in the myenteric plexus, which leads to regulation of gut motility, such as increased colonic transit time through the ENS.^70^ Similarly, alterations in the gut microbiome and its metabolites can induce activation of the HPA axis and associated immune responses.^71^ Studies in GF mice showed that the absence of gut microorganisms is linked to neuroendocrine and behavioral alterations.^72,73^ GF mice showed exaggerated HPA axis activation in response to stress, with increased levels of circulating corticosterone, and independent of cytokine-mediated pathways, as shown by unaltered IL-1β and IL-6 levels in the plasma. Intriguingly, colonization with specific strains, such as *B. infantis* or *E. coli*, modulated the HPA axis by either increasing or decreasing its activity, respectively.^73^

### 3.5 Microbially-derived neuroactive metabolites

The MGBA is regulated by a large number of different neurotransmitters, neuropeptides, and microbially derived products.^74^ While neurotransmitters have multiple effects on gut ecology, the microbiome itself produces and releases neurotransmitters. Studies showing a microbial origin for dopamine, norepinephrine, gamma-aminobutyric acid (GABA), and serotonin, among others, point toward a potential role of microbiota-produced neurotransmitters in influencing brain function, as discussed below.^75^ Additionally, neuropeptide synthesis is influenced by hormones and amino acid availability, which is controlled by the microbiome.^74^ However, neuropeptides can also modulate the composition and function of the gut microbiota, reflecting the complex bidirectional crosstalk that integrates these systems.

Bacteria have unique structural components known as microorganism-associated molecular patterns (MAMPs), such as LPS. MAMPs play an important role in host development and immune function.^76^ Transformation of host-derived components involves the production of secondary bile acids and steroid hormones, which have neuroactive properties.^77^ The gut microbiota also plays an important role in the transformation of dietary substrates. Many microbially-derived metabolites of amino acids, carbohydrates, and other plant-derived molecules exert pleiotropic effects on the MGBA and ultimately, on brain function and behavior. The microbial metabolism of tryptophan, tyrosine, and phenylalanine influences the production of neurotransmitters such as serotonin, noradrenaline, and dopamine.^78^ Tryptophan is particularly relevant to the brain, including tryptophan derivatives, indoles and kynurenine, which can modulate glutamate signaling and have been implicated in anxiety-like behavior and cognitive dysfunction.^79,80^ The microbiome is also implicated in the production of the major inhibitory neurotransmitter GABA, and alterations in glutamate/GABA circuits in the brain have been associated with the development of autism spectrum disorders, schizophrenia, major depressive disorder, and other neuropsychiatric disorders.^11^ For instance, a wide analysis of GABA production levels from several commercially available probiotics highlighted the strain-specific ability of *Levilactobacillus brevis* and *Lactiplantibacillus plantarum* in secreting GABA both *in vitro* and *in vivo*.^81^ Additionally, expression of GABA receptors can be modulated by specific bacterial strains such as *Lactobacillus rhamnosus* in mice, with data indicating a decrease in GABA_B1b_ mRNA in the hippocampus and amygdala, and concomitant decrease in GABA_Aα2_ mRNA expression in the prefrontal cortex. These changes were associated with reduced anxiety- and depression-like behaviors.^11^

Complex plant polysaccharides, or dietary fibers, are fermented by the gut microbiome to produce SCFAs, a class of compounds that has recently gained a great deal of attention because of their ability to influence multiple processes in the host, including behavior.^82,83^ SCFAs, such as butyrate, propionate, and acetate, are used as an energy source for colonic epithelial cells and can enter the systemic circulation and modulate the immune system through the regulation of gene expression.^84^ SCFAs can also cross the BBB via monocarboxylate transporter-expressed endothelial cells, where they can directly act on both neurons and microglia (as extensively reviewed in ref^84^)in the neurological and neuropsychiatric disorders investigated. Recent findings on the role of neuroactive metabolites and their effects on the MGBA^21,85–93^ are summarized in Table 1. In the following section, we will focus on the link between gut dysbiosis and neurological disorders in the context of the MGBA.

**Table 1.** Neuroactive metabolites and their effects.

Metabolite	Brain effects	Gut effects	Immune system effects	References
SCFAs Propionate, Acetate, Butyrate	Inflammation – Neuronal activation + Axonal damage – Treg differentiation + GABA/Glutamate in hypothalamus + Occludin + (in frontal cortex and hippocampus)	Colonic inflammation – (through GPCR binding on APCs)	Inflammation – (activation of GPRCs) Oxidative stress - (Cd41 expression on HCMEC/D3 cells downregulated)	^85–87^
Tryptophan metabolites Indoles, Kynurenine	Vagal stimulation +	Intestinal barrier function + Mucus production +	Generation of regulatory T-cells + (through Ahr receptor)	^21,85,88^
GABA	Vagal nerve firing +	Modulate intestinal motility and inflammation	Inflammation – (through decrease of T-cell activity)	^21^
Bile Acids	Elevated levels cause inhibition of hepatic glucocorticoid clearance, leading to HPA-Axis disruption	Intestinal absorption of lipids and vitamins +	T-reg cell differentiation + Th1/17 differentiation -	^90,91^
Serotonin	Receptors in hippocampus and neocortex support cognition and afferent signaling	Intestinal motility + 5-HT1A receptor degranulates enteric mast cells, histamine + Enterochromaffin cells +	Induces pro-inflammatory cytokines + T-cell activation	^92,93^

## 4. Microbiome changes with aging

Microbial colonization of the GI tract begins at birth, with mother-to-infant vertical transfer of skin and vaginal microbial strains, which are then replaced by species typically seen in the adult gut. The first 1,000 days of life are characterized by a relatively low bacterial diversity, with genus Bifidobacterium representing up to 50% of the infants’ gut microbial community, followed by an intense remodeling of the foundational gut microbiota.^94^ Fluctuations in the infant microbiome configuration follow the transition from a breast milk-based diet to an adult-like diet at the time of introduction of solid food and are also influenced by the surrounding environment.^95^ Given the instability of the infant gut microbiota, exposure to detrimental environmental factors can disrupt this highly orchestrated microbial succession, leading to gut dysbiosis that persists well beyond the early developmental period and serves as a risk factor for disease later in life.^96^ This first period of microbial colonization is characterized by rapid changes determined by either environmental factors or intrinsic ecological drifts which continue throughout adolescence. In contrast, relative stability in gut microbial communities is reached during adulthood. Even though environmental factors, such as antibiotic treatment and changes in diet, can alter gut microbiome composition, key species are thought to regulate the integrity and stability of the ecosystem in adult individuals.

Aging is a complex, time-dependent decline of the physiological, immunological, metabolic, and genomic functions in the host. Many studies^97^ have attempted to describe the molecular and cellular hallmarks of aging, which include cellular senescence, telomere dysfunction and damage, alterations in protein synthesis and epigenetic regulation, mitochondrial and nutrient-sensing dysfunction, and depletion of stem cell reserves. More recently, chronic inflammation and gut dysbiosis have emerged as additional factors associated with aging.^98^ Immune aging is characterized by “immunosenescence,” a progressive decline in the ability of both innate and acquired immunity to induce an effective response to both infection and vaccination.^99^ Changes in the gut microbiome are implicated in both age-related pathologies and potentially act as mediators of the aging process.^100^ A major change seen in the gastrointestinal tract with aging is a decrease in the barrier integrity of the intestinal epithelium.^2^ The function of the intestinal barrier can be modulated by commensal resident microbiota. *Akkermansia*, for example, given orally to mice has been shown to alleviate senescence-related phenotypes in the intestines of aged mice.^101^ In young mice, higher amounts of *Parabacteroides* and *Akkermansia* were found, whereas these two bacterial taxa decreased in the gut of aged mice.^101^ *Akkermansia* induces mucus production in the gut, which may help to restore and maintain barrier integrity.^102^ The effects of aging on the microbiome also contribute to changes in pathways controlled by the gut microbiome, including those involved in the biosynthesis of GABA and SCFA production, which are less enriched in aged mice than in young mice.^101^ In humans, *Bifidobacterium* are found at higher levels in infants, while *Lachnospiracae* levels are higher in adults.^103^ However, some consistencies remain throughout a healthy lifespan, evident through data that show how certain bacterial species, such as *Enterobacteriaceae*, are found at similar levels in both infants and the elderly.^103^ Some researchers have suggested that changes in the microbiota that occur with aging may be better assessed using biological age, rather than chronological age, as the contributing factor. Although methods for determining biological age can produce varying results, researchers have found that the Frailty Index (FI34), a method to calculate biological age, is better correlated with changes in microbiota in humans than chronological age.^104^ One method of identifying and characterizing biological age may be through characterization of the gut microbiome composition. As implicated in many studies^6,101,103,105,106^(Table 2), the gut microbiome composition is significantly altered with increasing age, even in the absence of an ND, which further exacerbates gut dysbiosis.

**Table 2.** Gut microbiota alterations in aging.

Microbiota Alterations	Analysis Method	Key Results	Potential Mechanism	Subjects	Reference
Clinical					
Decreases in Faecalibacterium, Odoribacter, and Dorea Increases in Clostridium species and Ruminococcus	Analysis study using shotgun and 16S rRNA gene amplicon-based sequencing profiles	Specific gut bacterial taxa are better markers of unhealthy aging than summary or diversity indices; healthy aging markers occupy core positions in the gut microbiome	No specific mechanisms proposed	Analysis of 21,041 gut microbiome profiles from ages 18 to 100+ years	^6^
Increases in Clostridiaceae and Megamonas	V3-V4 16s rRNA sequencing	The gut microbiota in subjects younger than 20 years changed with age as it matured, and that of subjects older than 70 years changed into an aged composition	No specific mechanisms proposed	367 total subjects; ages 0–104 years	^103^
Decreases in Lactobacillus and Oxalobacter (in extremely aged subjects)	16s rRNA sequencing	Age-related decline of the beneficial functions of gut microbiota, as well as increase of inflammation and disease, especially for people older than 90s.	No specific mechanisms proposed	371 total subjects, ages newborn to centenarian	^105^
Experimental					
Increases in Odoribacter, Clostridium, Porphyromonadaceae and Butyricimonas	V3-V4 16s rRNA sequencing	The cecal microbiota of mice is significantly altered with aging; perturbations of the MGBA, resultant of normal aging, may contribute to peripheral inflammation, altered anxiety behaviors and cognitive impairment	No specific mechanisms proposed	12 young (2 month old) and 10 aged (18 month old) male WT mice	^106^
Decreases in Akkermansia, Parabactreoides Increases in Helicobacter, Turicibacter, and Prevotella	16S ribosomal RNA and metagenomic sequencing	Age-associated changes are present in the compositional structure of the gut microbiome and its function Rejuvenation models (i.e. through parabiotic pairing of an aged mouse with a young mouse) restore age-dependent alterations of intestinal function	Akkermansia administration restored intestinal integrity by activating epithelial cells, supporting the growth of other beneficial commensals	3 models of aging: co-housing (4 and 18 month old mice), serum injection (5 month and 20 month old mice treated with aged serum), and parabiosis (aged mice paired with young mice or age controls)	^101^

## 5. The role of an aging maternal gut microbiome in offspring neurodevelopment

**Figure 3.**
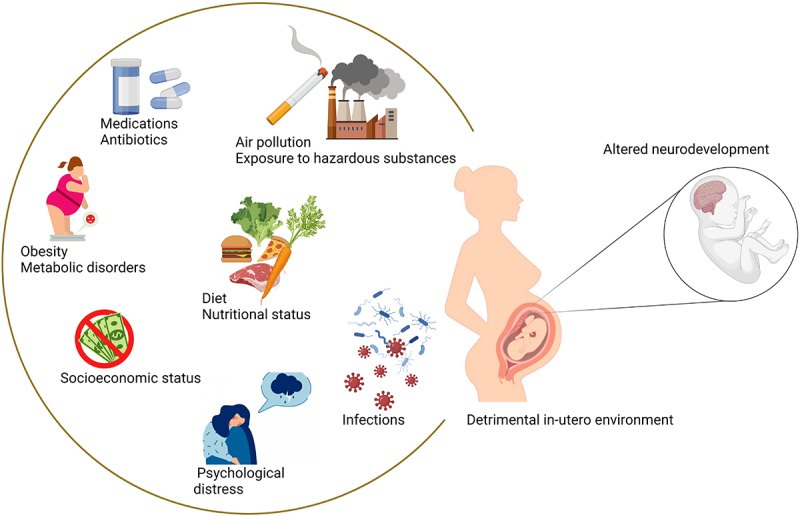
Environmental factors causing detrimental alterations in the maternal exposome. Toxin and pollutant exposure, infection during pregnancy, diet and metabolic status, smoking, psychosocial stressors such as low socioeconomic status, major life events, and pregnancy-related stressors, can determine broad changes in the maternal environment, thereby jeopardizing pregnancy outcomes and fetal developmental programming. Created in Biorender.com.

The maternal exposome^107^ in particular, plays a crucial role in early life development.^108^ Detrimental alterations in the maternal exposome can trigger fetal programming events that predispose offspring to chronic health conditions, including brain disorders, later in life (‘Developmental Origins of Health and Disease’^109^) (Figure 3). Multiple studies in the context of maternal obesity, infections, and antibiotic use during pregnancy have confirmed the crucial role of maternal gut dysbiosis as a mediator of offspring’s neurodevelopment.^110–112^ Interestingly, aging-associated alterations in gut microbiome,^113^ which are transmitted to the offspring, also result in chronic dysbiosis and increased disease risk in adult offspring. Additional epigenetic programming during early development can also be passed on to subsequent generations.

Epidemiological studies have linked advanced maternal age (AMA; ≥35 years) to adverse pregnancy outcomes, such as diabetes and preeclampsia,^114^ and an increased risk for metabolic and brain disorders in the offspring.^115–119^ This may involve epigenetic reprogramming in either the oocyte^120^ or the fetus, or direct effects of inherited dysbiosis. The precise mechanisms by which AMA affects brain development are unclear.^114^ Over the past three decades in the United States, there has been a steady increase in the birth rate for women aged 35–39 years from 45.9 per 1000 women in 2010, to 52.7 in 2019.^121^ Similarly, the birth rate for women aged 40–44 years rose by 5% from 2020 to 2021.^122^ Although a multitude of factors contribute to the increased risk of complications seen in older mothers and their offspring, recent studies have implicated changes in the maternal microbiome that may contribute to these poor outcomes.^123,124^

**Figure 4.**
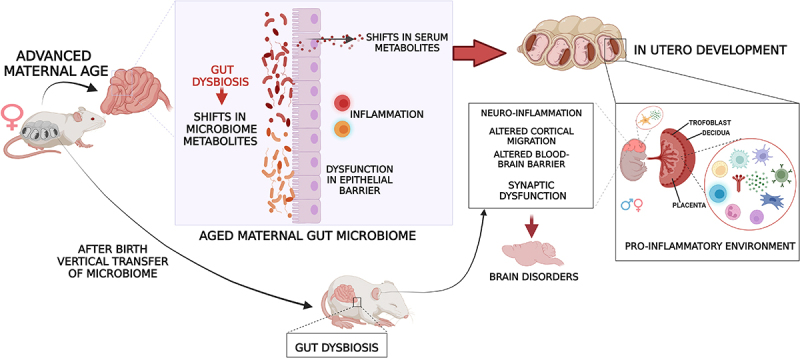
Proposed mechanism for AMA-related fetal programming and increased risk for brain disorders in offspring. AMA-associated gut dysbiosis and increased inflammation may drive abnormal immune activation in both the placenta and the fetal brain, specifically in microglial cells. Therefore, increased brain inflammation could alter neurodevelopment through multiple mechanisms, including epigenetic modifications in neuronal and glial cells. At birth, vertical transmission of a dysbiotic gut microbiome sustains this systemic neuroinflammation in the neonate, jeopardizing postnatal neurodevelopment and adult health. Created in Biorender.com.

Despite the established association between aging, gut dysbiosis, and increased inflammation, few studies have focused on the effects of maternal age-related gut dysbiosis on fetal development and brain health outcomes. A recent study in humans showed that both the vaginal and the gut microbiomes of women displayed significant differences in microbial composition based on age and pregnancy status.^125^ Given that aging is characterized by gut dysbiosis, an altered intestinal metabolome, increased barrier permeability, and chronic, low-grade inflammation, it is possible that advanced maternal age could alter both the maternal gut microbiome and gut mucosal and systemic immune system, similar to what is observed in maternal obesity. This can then disrupt physiological adaptations to pregnancy and impair placental function, leading to increased brain and systemic inflammation in the fetus and alterations in neurodevelopment (Figure 4). These effects might be mediated by gut dysbiosis-mediated alterations in the abundance of microbiome-derived metabolites, such as SCFAs,^126–128^ which can (1) actively modulate maternal immune cells, leading to enhanced systemic and placental inflammation^129^ and (2) cross the placenta and directly influence epigenetic programming of fetal brain cells, neuroinflammation through microglial activation, and neural circuit formation. After birth, maternally inherited dysbiosis can sustain systemic and neuroinflammatory events in offspring, leading to detrimental effects on brain function and behavior. It is unknown how long these detrimental changes last in the offspring, or if these early developmental events can alter the risk for neurodegenerative diseases later in life. Longitudinal studies are required to address this question.

## 6. Neurodegenerative diseases

### 6.1 Parkinson’s disease (PD)

PD is a neurodegenerative disease characterized by a loss of dopaminergic neurons in the substantia nigra pars compacta and the deposition of insoluble alpha-synuclein polymers in neurons, forming Lewy bodies.^130^ Approximately 80% of PD patients suffer from GI dysfunction.^131^ PD patients commonly suffer from symptoms such as constipation, which precede the clinical diagnosis of PD and its other hallmark symptoms such as bradykinesia and dementia,^131^ indicating that gut dysfunction may play a role in the pathogenesis of PD. Recent studies have attempted to identify specific microbiota changes that may lead to PD. An MPTP-induced mouse model of PD showed very distinct changes in the gut microbiome, including a significant decrease in the levels of *Prevotella* and *Faecalibacterium* and an increase in *Ralstonia* bacteria, compared to control mice.^132^ Additionally, *Enterobacteriaceae* is increased in both humans and rodent models of PD.^133^ The specific mechanisms behind these microbiota changes and their contribution to PD are yet to be understood. However, certain bacterial species present in the gut or fecal matter of patients with PD have been well-described (Table 3). *Citrobacter rodentium*, which is enriched in patients with PD, has also been shown to aggravate motor symptoms in mouse models. Increases in *Proteus mirabilis* are also linked to PD symptoms and have been shown to promote motor deficits in mouse models of PD.^139^ Researchers have linked the changes in the expression of these specific bacterial genes to mechanisms that regulate lipid biosynthesis and secretory pathways, including dopamine (DA) regulation and production, as many PD symptoms can be traced back to a decrease in DA levels.^140^ Studies have shown that some of the bacterial enzymes residing in the gut produce DA,^141^ further solidifying the link between the roles of microbial-derived metabolites in the progression of PD. This direct correlation between bacterial metabolites and the onset of PD symptoms implies there a role of the MGBA in the pathogenesis of PD (Table 3)^134–138;^ however, the specific metabolites involved remain to be studied in-depth.

**Table 3.** Gut microbiota alterations in Parkinson’s disease (PD).

Microbiota Alterations	Analysis Method	Key Results	Potential Mechanism	Subjects	Reference
Clinical					
Decreases in Prevotellaceae, Faecalibacterium, and Lachnospiraceae Increases in Ruminococcaceae, Verrucomicrobiaceae, and Bifidobacteriaceae	Meta-analysis; inclusion criteria list fecal samples	Shared alterations of certain gut microbiota are present in PD patients across different geographical regions	Alterations in specific microbiota levels contribute to the pathogenesis of PD; abnormalities in gut microbiota and its metabolic products may be triggers for the formation of Lewy bodies in PD	959 patients with PD, 744 healthy controls; ages 62 to 76.5 years	^134^
Decreases in Lachnospiraceae and Faecalibacterium Increases in Akkermansia, and Collinsella	16S rDNA V4 amplicon sequencing and qPCR of fecal samples; serum metabolomics	Alterations in PD are most pronounced at the level of coabundant bacterial clusters; PD patients had significantly lower SCFA-producing bacteria levels; taxonomic differences in the gut composition of PD patients	Decrease in butyrate production contribute to disrupted colonic motility. Alterations found in the enrichment of fucose degradation pathways, which may cause breakdown of the intestinal mucosal layer in PD and hinder the process of proteolytic metabolite generation	197 PD and 103 control subjects; ages 40 to 85 years	^135^
Decrease in Lachnospiraceae, and Butyricoccus Increase in Akkermansia	16s sequencing of fecal samples	Gut microbiome alterations are already present in PD patients prior to treatment with dopaminergic medication	No specific mechanism proposed; In pre-treatment PD subjects: overall dopaminergic input may influence gut microbiome composition via cerebral signaling or modulation of stool transit times	56 PD and 87 control patients; ages 64 to 67 years	^136^
Experimental					
Decrease in Lachnospiraceae, Proteobacteria Increases in Prevotellaceae and Akkermansia	V3–V4 regions of bacterial 16S rRNA gene were amplified using PCR	MPTP affects the composition of gut microbiota and damages the intestinal barrier in mice	No specific mechanism proposed	20 MPTP PD-model mice, 20 control mice	^137^
Decreases in Lachnospiraceae and Butyricicoccus	V4 16s rRNA sequencing	PD causes gut dysbiosis in mice colonized with Pd-microbiota and PD-derived gut microbiota promote motor dysfunction	aSyn aggregation by the inflammatory environment found I the gut of PD patients may be related to activation of microglia which enhances inflammatory PD pathology; may implicate potential PD therapies based on targeting inflammation via the gut	ASO mice and WT control mice	^138^

### 6.2 Alzheimer’s disease (AD)

One of the most common diseases associated with aging is AD; increasing age is the greatest risk factor for late-onset AD.^142^ Although some treatments targeting amyloid clearance in AD patients have emerged, the availability of therapeutics targeting the prevention of amyloid development is limited.^143^ Interestingly, recent studies have demonstrated that amyloid-beta (Aβ) plaque deposition is linked to the composition of the gut microbiota. Studies by our research group have shown that in a Tg2576 transgenic mouse model of AD, gut inflammation and dysbiosis precede the accumulation of amyloid plaques in the brain,^144^ indicating that gut dysbiosis may play a role in the development of amyloid pathology, although these findings require further validation in additional animal models and in AD patients. In the same study, we found Aβ deposition in postmortem gut samples from patients with AD pathology, which suggests that gut-derived Aβ is associated with AD pathophysiology in some ways. Ongoing studies are exploring specific changes in the microbiome to discover a link between AD and the MGBA. Several studies have demonstrated that the composition of gut microbiota is altered in patients with AD. Vogt et al. found that Firmicutes and Bifidobacterium are decreased and *Bacteroidetes* are increased in elderly AD patients (age:71.3 ± 7.3 years) compared to age-matched controls (age:69.3 ± 7.5 years).^145^ Liu et al. also observed a decrease in Firmicutes and an increase in Proteobacteria in the elderly patients.^146^ In addition, Cattaneo et al. showed that the abundance of pro-inflammatory bacteria such as Escherichia/Shigella is increased in patients, whereas that of an anti-inflammatory bacterium (e.g., E. rectale) is increased.^147^ These findings indicate that gut microbiota may be associated with AD pathophysiology.^148^ Other studies (Table 4)^145,146,149–152^ show that among different species and disease models of AD, many of the mechanisms or changes governing microbiota-driven alterations in AD development are similar. Some of these changes, however, appear to be species-dependent, that is, they are observed differently in humans and animal models.

**Table 4.** Gut microbiota alterations in Alzheimer’s disease (AD).

Microbiota Alterations	Analysis Method	Key Results	Potential Mechanism	Subjects	Reference
Clinical					
Decreases in Actinobacteria (Bifidobacterium) Increase in Bacteroides	16s rRNA sequencing of fecal samples and PICRUSt metagenomics analysis	AD patients have reduced microbiome richness compared to healthy controls	Increased abundance of gram-negative bacteria (Bacteroides) in AD patients causes increased translocation of LPS from the gut to systemic circulation, which exacerbates AD pathology through inflammatory pathways	50 subjects, ages 69–71 years; 25 AD and 25 control	^145^
Decreases in Bacteroides, Dorea, and Faecalibacterium	16s rRNA sequencing of fecal samples	A significant negative correlation is observed between the severity of gut barrier dysfunction and cognitive function in AD and MCI patients	Increased production of SCFA’s by the microbiota enriched in control patients compared to AD patients elucidates a mechanism through which decreases in butyrate-producing bacteria, correlated with AD progression, limits anti-inflammatory effects in the gut exacerbating AD symptoms	97 subjects, ages 50–85 years; AD, MCI, and control groups	^146^
Decrease in Ruminococcus, Butyricimonas, and Oxalobacter Increase in Flavonifractor	16S rRNA sequencing of fecal samples	Gut microbiome alterations precede onset of clinical AD symptoms, independently of the influence of cognitive impairment	Specific mechanism not proposed	31 MCI patients; 65 healthy controls, ages 65+	^149^
Experimental					
Decreases in Bifidobacterium, Lactobacillus, Firmicutes	16s rRNA qPCR analysis of colonic flushings	Significant microbiota alterations and gut barrier dysfunction in AD mice compared to non-Tg controls	Gut microbiota modulate peripheral inflammatory pathways through inflammasome (NLRP3) signaling that contribute to CNS neuroinflammation and subsequent neurodegeneration	5×FAD mice, ages 5 and 15 months Non-Tg WT controls	^150^
Specific bacterial taxon alterations not reported	454 pyrosequencing of 16S rRNA gene amplicons, on fecal metagenomic DNA	Significant community-level microbiota alterations and altered colonic gene expression in AD mice; FMT-induced modifications toward the microbiota pattern of WT mice ameliorated amyloidosis, tau pathology, reactive gliosis and cognitive impairment in ADLP^APT^ mice	The downregulation of genes related to mitochondrial and ribosomal activities in ADLP^APT^ mice may lead to aberrations in ATP synthesis and protein synthesis, key features of NDs	ADLP^APT^ mice, age 8 months	^151^
Decreases in Actinobacteriota, Verrucomicrobiota Increases in Proteobacteria	16S rRNA sequencing of fecal samples	Microbial diversity is reduced in patients with advanced AD	Increased abundances of pro-inflammatory bacteria in AD patients exacerbate and trigger AD symptoms prior to onset of apparent cognitive impairment	3×Tg-AD mice; 3-, 6- and 9-month-old aged-matched WT control mice	^152^

To understand the role of gut microbiota in AD, microbiota-targeted interventions, including fecal microbiota transplantation (FMT), have recently been employed in animal models of AD. Sun et al. performed FMT from naïve WT mice (6 months old) into age-matched APPswe/PSEN1dE9 transgenic mice. These transgenic mice exhibit occasional Aβ deposits by six months and abundant plaques by nine months.^153^ Cognitive impairment is seen at 12–13 months in this mouse model.^153,154^ They found that FMT improves cognitive function and synaptic plasticity, and decreases levels of Aβ40, Aβ42, and p-Tau231 in the brain of recipient mice.^155^ The beneficial effects of FMT seen in the recipient mice were associated with higher levels of fecal SCFAs, such as butyrate. More recently, Kim et al. transplanted the fecal microbiome of 5×FAD mice into WT mice.^156^ Compared with many other models, 5×FAD mice show more rapid Aβ deposits in the brain (<3 months) and cognitive impairment (<6 months).^157,158^ They showed that reconstitution of the 5×FAD microbiome reduced spatial learning and memory in recipient WT mice, compared with recipient mice treated with the biome from WT mice. In addition, recipient mice with the 5×FAD microbiome showed decreased neurogenesis, increased neuroinflammation including microglial activation, and elevated pro-inflammatory cytokines (e.g., TNF-α and IL-1β) in the brain. Interestingly, the recipient mice had increased levels of both pro-inflammatory cytokines (e.g., TNF-α, IL-1β, and IL-6) and anti-inflammatory cytokines (e.g., IL-10) in the colon, whereas only IL-1β, but not the other tested cytokines, was increased in the plasma. This indicates that the reconstitution of the gut microbiota with healthy microbiota can ameliorate memory dysfunction by regulating inflammation in AD mice through the MGBA. It has also been reported that transferring healthy microbiota into ADLP^APT^ mice, a mouse AD model with both amyloid and neurofibrillary tangle pathology, significantly reduces the formation of amyloid plaques and tangles, resulting in cognitive improvement.^151^ Taken together, these findings indicate that the restoration of a healthy biome can delay the symptoms and progression of AD in animal models. Thus, future investigations of the role of gut microbiota as new therapeutic targets for AD are warranted.

### 6.3. Cerebral amyloidosis and cerebral amyloid angiopathy (CAA)

Amyloidosis, one of the most significant pathologies found in AD, is also affected by gut dysbiosis in related neurodegenerative disorders, including CAA. CAA is a small vessel disease characterized by amyloid deposition in the basement membrane of the brain vasculature.^159^ Aging is a major risk factor for CAA; CAA leads to progressive cognitive impairment in elderly patients, and also contributes to ischemic small vessel disease and intracerebral hemorrhage.^160^ In a mouse model of APP and PS1 mutations (APP/PS1 mice), Chen et al. showed that the microbiota composition between APP/PS1 and WT mice diverged significantly at 1–3 months of age, prior to the onset of cognitive symptoms, amyloid deposition, and neuroinflammation (e.g., microglial activation) in the brain.^161^ This study, consistent with many others, demonstrated that higher levels of *Enterobacteriaceae*, as well as *Verrucomicrobia* were present in the gut of mice that developed amyloid plaques when compared to control mice.^161,162^ There are very limited studies demonstrating the regulatory role of gut microbiota in CAA or cerebral amyloidosis. Therefore, the investigation of how vascular Aβ versus parenchymal Aβ affects the gut microbiota and gut dysbiosis-associated cognitive impairment in the context of CAA or cerebral amyloidosis will be an important future direction.

## 7. Acute neurological injuries

### 7.1 Stroke

Stroke is a leading cause of mortality and morbidity in elderly patients. Options for acute treatment such as recombinant tissue plasminogen activator and endovascular thrombectomy are available,^163^ however post-stroke treatment is critical as chronic disability and other long-term health consequences of stroke persist for decades.^164^ The majority of strokes are caused by occlusion of an artery, either by an embolus or an *in-situ* thrombosis, leading to an area of brain ischemia.^165^ Interestingly, recent advances in metagenomics have revealed that stroke remarkably alters the composition of the microbiome, and in turn, this stroke-induced “gut dysbiosis” can exacerbate neuroinflammation and behavioral deficits in a mouse model of stroke.^16,166–168^ In a study comparing young, stroke mice to uninjured aged controls, we found that the gut microbiome of stroke mice is altered and resembles the microbiome composition of uninjured aged mice.^169^ It was previously reported that post-stroke translocation of gut microbes into the lung leads to sepsis in mice.^170^ In other mouse model studies of stroke, some of the more specific microbiota changes have been characterized.^126,166–168^ Using mouse models of ischemic stroke, such as middle cerebral artery occlusion (MCAO), Singh et al. found that stroke can cause gut dysbiosis, as assessed by reduced bacterial diversity and Bacteroidetes overgrowth, which were associated with impaired gut integrity and motility.^166^ They subsequently transplanted post-stroke microbiome into GF mice. Interestingly, recipient GF mice had larger infarct volumes and worse behavioral deficits, along with increased pro-inflammatory T cells in both the intestines and the ischemic brain. Furthermore, Benakis et al. revealed that gut microbiota can regulate T cell trafficking from the gut into the leptomeninges after stroke and that specific types of T cells, such as regulatory T (T_reg_) cells and IL-17^+^ γδ T cells, are critical in regulating neuroprotection by modulating gut-to-brain signaling following stroke.^168^

As the elderly are more prone to stroke than younger populations, our research group has focused on the regulatory role of MGBA and the underlying mechanisms of stroke in aged mice. We first examined whether aged mice were more susceptible to stroke-induced gut permeability and bacterial translocation than young mice. Aged mice had increased gut permeability after stroke and higher mortality compared to young mice. When we orally gavaged young and aged mice with GFP-tagged *E. coli*, aged mice exhibited increased bacterial translocation into peripheral tissues, such as the mesenteric lymph nodes, compared with young mice,^171^ indicating the direct effect of aging on gut dysbiosis and bacterial translocation after stroke.

To profile the effect of aging on the composition of the gut microbiota, we performed 16S rRNA-seq on fecal samples from young and aged mice. We found that the composition of the gut microbiota is distinct between young and aged mice; the Firmicutes: Bacteroidetes ratio was higher in the aged biome compared to the young biome, indicating age-induced gut dysbiosis. Next, we transplanted the aged microbiome and young microbiome into young and aged mice, respectively, using FMT prior to stroke (transient MCAO). Interestingly, young recipient mice with aged microbiome showed increased post-stroke mortality and functional deficits. Conversely, aged recipient mice transplanted with young microbiome showed better post-stroke outcomes.^169^ Although we found that preconditioning of the aged gut using a young microbiome prior to stroke can contribute to post-stroke recovery, stroke is not predictable. Therefore, in a separate study, we transplanted young microbiome into aged mice several days after stroke (as a treatment) to determine whether post-stroke FMT can improve post-stroke recovery.^126^ We found that post-stroke reconstitution of young microbiome significantly improved functional outcomes (e.g., increased spontaneous locomotor activity and cognitive functions, and reduced depressive-like phenotype) along with decreased inflammation in both the brain and gut. Post-stroke, young FMT increased T_reg_ cells in the small intestine and enhanced protective mucus production in the large intestine. Moreover, aged stroke mice with young FMT had higher T_reg_ cells and lower IL-17^+^ γδ T cells in the ischemic hemisphere than aged stroke mice with aged FMT. Using metabolomic analysis, we revealed that the young microbiome contains higher levels of SCFAs, such as acetate, butyrate, and propionate. Based on our metagenomic data, we selected four SCFA producers (*Bifidobacterium longum*, *Clostridium symbiosum*, *Faecalibacterium prausnitzii* and *Lactobacillus fermentum*) and orally gavaged aged mice with these, and the pre-biotic inulin, after stroke. Interestingly, post-stroke bacteriotherapy using SCFA-producers and inulin increased SCFA levels in the gut, plasma, and brain, and synergistically improved post-stroke recovery by reducing IL-17 production in γδ T cells in the brains of aged stroke mice. In a follow-up study, we found that the aged microbiome alone is sufficient to produce cognitive decline in young GF mice compared to the young microbiome. In conclusion, our findings suggest that aging should also be considered as a detrimental factor regulating the MGBA in stroke. Several other studies^166,172–175^(Table 5) suggest that the gut microbiome is an essential regulator of post-stroke recovery, and the identification of specific bacterial populations after stroke may uncover various mechanisms through which the microbiome influences inflammation post-stroke.

**Table 5.** Gut microbiota alterations in stroke.

Microbiota Alterations	Analysis Method	Key Results	Potential Mechanism	Subjects	Reference
Clinical					
Decreases in Lachnospiraceae and Ruminococcaceae Increases in Enterobacteriaceae, Veillonellaceae (opportunistic pathogens), Bifidobacterium, and Lactobacillus (lactate-producers)	16S rRNA gene amplicon next-generation-sequencing and gas chromatography (SCFAs) on fecal samples	Participants at higher risk of stroke were characterized by the enrichment of opportunistic pathogens, low abundance of butyrate-producing bacteria, and reduced concentrations of fecal butyrate	No specific mechanism proposed	141 subjects ages 60+ years; low-, medium- and high-risk groups for stroke	^172^
Decrease in Bacteroides Increases in Akkermansiaceae, Fusobacteriota, Desulfobacterota, Ruminococcaceae, and Oscillospirales	16S rRNA sequencing and gas chromatography (SCFAs) on fecal samples	Poststroke subjects harbor an altered gut microbiota composition. SCFAs may play a significant role as key mediators in the modulation of pain in poststroke patients	No specific mechanism proposed;	20 stroke patients and 20 healthy controls, ages 18–80 years	^173^
Decrease in Ruminococcaceae Increases in Proteobacteria and Gordonibacter	Two-sample Mendelian randomization analysis to test the causal relationship between gut microbiome and stroke subtypes	There is a causal effect of the abundance of specific bacterial features on the risk of certain stroke subtypes (large artery, small vessel, and cardioembolic)	No specific mechanism proposed	Genetics from 18,430 subjects, stroke gene data on 40,858 cases from 3 subtypes of stroke	^174^
Experimental					
Bifidobacterium, Bacteroides, Prevotella, Clostridia, and Faecalibacterium are significantly altered post-stroke	16s rRNA V1-V3 amplicon sequencing on mouse fecal samples	Stroke induces intestinal microbiota dysbiosis and reduces species diversity in the gut microbiome composition Fecal microbiota transplant from control mice is neuroprotective after stroke	Post-stroke dysbiosis favors predominant expansion of proinflammatory T-cell subpopulations, T cell priming in stroke and the role of gut-resident T cells	WT and GF mice	^166^
Increase in Enterobacteriaceae	16s RNA sequencing	Stroke mice receiving FMT from stroke patients present higher Enterobacteriaceae abundance and lower fecal butyrate levels than control mice	Enterobacteriaceae, or other butyrate-producing bacteria may be involved in rhANP-mediated changes in the gut microbiota which contribute to post-stroke pathology	WT mice with MCAO; 83 human fecal samples	^175^

### 7.2. Traumatic brain injury

**Figure 5.**
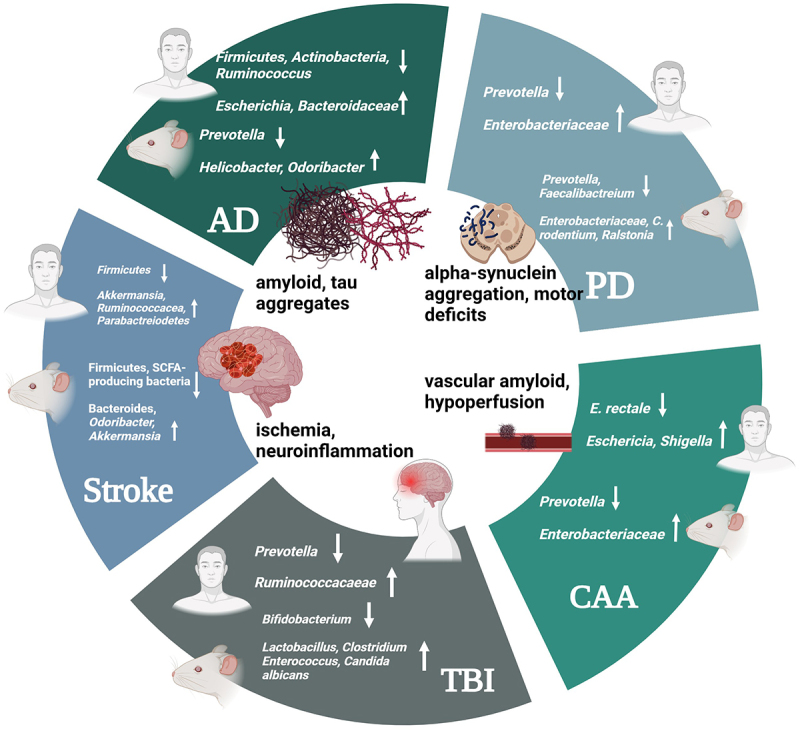
Major symptoms and features of NDs are accompanied by multiple alterations in specific microbial species, changes which can be consistent or contradictory between the human microbiome and mouse models. Created in Biorender.com.

Approximately 2 million people sustain a head injury annually in the United States.^176^ Along with many other symptoms of traumatic brain injury (TBI), intestinal dysfunction has emerged as a chronic consequence of head injury. Studies in rat models of TBI show a loss of alpha diversity and alterations in bacterial taxa that reside in the gut.^177^ These changes have also been observed in human fecal samples collected from athletes or trauma patients who sustain head injuries or concussions. One such study involving football players who had concussions demonstrated changes in specific bacterial species, such as *Agathobacter* and *Ruminococcaceae*, when compared to healthy, uninjured control subjects.^178^ Similar results were found in other studies^177–180^ and are reviewed in Table 6. The effect of TBI on the gut microbiome is immediate. The intestinal microbiota becomes disrupted within hours of injury and can lead to chronic inflammatory processes.^181^ The acuity of post-TBI gut microbiome changes provides evidence that microbiome-targeted therapies could be beneficial for TBI and related head injuries. Therapies targeting microbial alterations that occur with TBI could alleviate the chronic effects of the injury by limiting downstream consequences at the start of injury progression. A summary of the potential role of the gut microbiota in NDs is provided in Figure 5.

**Table 6.** Gut microbiota alterations in traumatic brain injury (TBI).

Microbiota Alterations	Analysis Method	Key Results	Potential Mechanism	Subjects	Reference
Clinical					
Decreases in *Prevotella* and *Bacterioidies* Increases in *Ruminococcaceae, Actinobacteria*, and *Verrucomicrobia*	16s V4 rRNA sequencing on fecal samples	Fecal microbiome composition was altered in the chronic TBI cohort compared to controls	Intestinal microenvironment alterations associated with TBI may be linked to alterations of amino acid metabolism in TBI patients	22 chronic, moderate-to-severe TBI patients and 18 healthy controls	^179^
Decrease in *Lachnospiraceae* Increase in *Ruminococcaeae*	16s rRNA sequencing	The overall alpha diversity of the microbiome composition did not differ between the time point groups; in concussed TBI patients, some bacterial taxon were significantly altered compared to non-concussed athletes	Processes related to the synthesis and degradation of sugars and aromatic compounds were among those showing the most significant fold changes in TBI; specific mechanisms yet to be proposed	33 male football players, ages 18–23 years; grouped as mid-, post-, and off-season	^178^
Experimental					
Decreases in *Ruminococcaceae* Increase in *Verrucomicrobiaceae* and *Bacteriodaceae*	Fecal bacterial 16S rRNA gene analysis	Alerted microbiome composition is observed in TBI mice, and the local and peripheral immune infiltration in GF mice post-FMT is altered	Gut microbiota control post-TBI hippocampal neurogenesis and its association with microglial morphology and T cell infiltration changes after injury; specific mechanistic link yet to be reported	GF and WT mice; cortical controlled impact (CCI) model of TBI	^180^
Decreases in *Bacteroidetes, Faecalibacterium*, and *Agathobacter* Increases in *Prevotella* and *Helicobacter*	V3-V4 16s rRNA sequencing	Microbiome composition and metabolic functions were altered in rats receiving TBI, the most pronounced of these alterations was a decrease in *Agathobacter* (butyrate-producer)	No specific mechanism proposed	25 3–4 month-old rats; focal open severe brain trauma model of TBI	^177^

## Conclusions and future directions

Considerable progress has been made in our understanding of the role of gut microbiota and their metabolites in health and disease. In this review, we have summarized key findings demonstrating the regulatory role of microbiota in neurodevelopment, neuroinflammation, and behaviors of the host, specifically in aging and age-related NDs. Although some changes in the gut microbial composition vary depending on the context, and substantial limitations (e.g., discrepancy between preclinical animal studies, differences in the gut microbiota composition between animals and humans, and variations in microbiome sequencing and bioinformatic pipelines) still remain, it is accepted that gut microbiota and metabolites are targetable, suggesting that there are novel therapeutic options for NDs through manipulation of the MGBA^155,182–187^ (Table 7). Of note, the microbiome has the potential to transform preventative care and reduce medical costs by enabling individual therapies in the field of precision medicine.^188,189^ Future studies will highlight bacterial strains, metabolites, and immune factors that might help identify new cellular and molecular targets for diagnostic tools and microbiome-targeting therapeutic and preventative approaches.

**Table 7.** Microbiome-targeted treatments for NDs.

Treatment Design	Key Results	Reference
Clinical		
**Probiotic treatment;** 169 middle-aged and older adults with MCI randomized to either probiotic (*Lactobacillus rhamnosus*) or placebo treatments for 3 months	Probiotic supplementation led to a decrease in the levels of *Prevotella* (which is enriched in MCI subjects prior to treatment) and this decrease was associated with an improved cognitive score	^182^
**FMT;** 36-week clinical trial including 34 PSP-RS patients (receiving healthy donor FMT) and 34 placebo controls (receiving saline)	The group receiving FMT had significantly improved symptoms of depression and anxiety, in addition to increases in levels of butyrate-producing bacteria (*Faecalibacterium*) post-FMT	^183^
**Dietary intervention;** NU-AGE diet randomized trial, 1279 older adults were included and divided into control or intervention groups, adhering to the diet for 1-year	Participants with higher adherence to the NU-AGE diet (Mediterranean-like diet) showed significant improvements in cognition and episodic memory compared to those with lower diet adherence	^184^
Experimental		
**Dietary intervention;** 2-month administration of either soluble fiber diet or control diet in a mouse model of AD	AD mice receiving the soluble fiber diet displayed lower memory impairments and anxiety than WT mice receiving the control diet. Additionally, intestinal morphological alterations were reduced in AD mice receiving the fiber diet, an effect accompanied by restoration of butyrate and propionate production in the gut content	^185^
**FMT;** 4-weeks of FMT administration from WT control mice to an aged mouse model of AD	FMT from age-matched WT control mice alleviated cognitive deficits and reduced the deposition of amyloid-beta in AD mice; FMT also reversed the alterations in specific microbiota changes seen in the AD mouse model prior to FMT	^155^
**FMT;** 2-weeks of FMT from control donor mice to a rotenone-induced model of PD mice	PD mice receiving FMT from control donors displayed alleviated motor symptoms and better gastrointestinal function, and restores blood-brain-barrier impairment	^187^
**Probiotic treatment;** oral administration of *Bifidobacterium breve* MCC1274 in WT mice	Thea administration of the B. breve MCC1274 probiotic decreased Ad-like pathologies in WT mice; soluble hippocampal amyloid-beta levels and neuroinflammation were decreased in mice receiving the treatment	^186^
